# Direct Restorations, Endodontics, and Bleaching: Materials and Techniques Used by General Dentists of New Zealand

**DOI:** 10.1155/2019/6327171

**Published:** 2019-03-07

**Authors:** Carolina Loch, Jithendra Ratnayake, Arthi Veerasamy, Peter Cathro, Robert Lee, Paul A. Brunton

**Affiliations:** University of Otago, Faculty of Dentistry, 310 Great King Street, Dunedin 9016, New Zealand

## Abstract

**Background:**

To investigate the selection and use of direct restorative materials, endodontic techniques adopted, and approaches to bleaching by general dentists in New Zealand.

**Methods:**

A questionnaire comprising 19 sections and 125 questions was distributed via mail to 351 general dentists in New Zealand who were selected, at random, from the Dental Council of New Zealand's 2016 register.

**Results:**

A total of 204 questionnaires were returned, of which 188 were usable. Direct resin composite was the most commonly used material for occlusoproximal cavity restorations in premolars (93.7%) and permanent molars (85.2%). Resin-modified glass ionomer cements (34%) and resin composite materials (31.4%) were more commonly used in the restoration of deciduous molars. Home-based vital bleaching was provided by a significant number of dentists (86%), while only 18% provided practice-based bleaching. Cold lateral condensation was the most commonly used obturation technique (55.8%), and 83% of respondents reported using rubber dam for treatments.

**Conclusions:**

The findings from this study indicate that dentists in New Zealand are adapting to new materials and technologies to provide high quality care to their patients. Aesthetic treatments such as bleaching have become an integral part of general dental practice.

## 1. Introduction

Dentistry is a profession where the care provided should be driven by evidence-based practice [1–3]. Conservative dentistry, which focuses mainly on the management and preservation of natural teeth, is still the major component of general dental practice. Traditionally, functional considerations have been the main drivers considered when restoring teeth. However, with the increasing power of media, computerization, and social reform, aesthetic dentistry has become a fundamental part of contemporary clinical dentistry [4, 5]. The development of new restorative materials and techniques has revolutionized contemporary dentistry, with patients demanding not only improvement to their dental appearance but also improvement in their overall facial and dental aesthetics [6].

There has been a vast paradigm shift in endodontic treatments over the past decade [7, 8]. Traditional endodontic treatments mainly focus on eliminating microorganisms from the entire root canal system, which offers patients the opportunity to maintain their natural dentition [9]. As the population ages, the demand of endodontic therapy can be expected to increase as patients seek dental options to keep their teeth for life. Conventional endodontic diagnosis traditionally has relied on two-dimensional radiographic images to deduce the extent and location of the root canal system [10]. New treatment approaches and technologies using modern state-of the-art equipment, instruments, and biocompatible materials have helped dentists to perform complex endodontic procedures more effectively and efficiently than ever before [11, 12]. Several changes have also occurred in the development and availability of restorative materials for children. The restoration of primary incisors which are severely decayed is one of the major challenges in paediatric dentistry. In recent years, paediatric dentists have new options of restorative materials and techniques able to provide efficient, durable, and functional restorations [13].

Evidence-based dentistry implies that dentists should choose techniques and materials based on research findings where robust evidence exists. This is one of the three papers reporting the findings of a recent investigation into general dental practice in New Zealand. The first of the current series of papers reported on demographic data and practicing arrangements [14]. The second paper primarily reported on indirect restorations and fixed prosthodontics [15]. The current paper investigates techniques, materials, and procedures adopted by general dentists in New Zealand with regard to direct restorations, bleaching, endodontic, and paediatric dentistry practice.

## 2. Methods

Ethical approval for this research was obtained from the University of Otago Human Ethics Committee (approval number D16/098). A questionnaire compromising 19 sections and 125 questions was sent by post in 2016 to a sample of 351 dentists who were selected from the 2016 Dental Council of New Zealand's register. Sampling was done proportionally to the number of dentists registered in each New Zealand region. The questionnaire was sent with a covering letter, $5 coffee voucher, and a stamped addressed envelope for return. After a period of four weeks, an email reminder was sent to all the dentists who did not respond.

Data from the returned questionnaires were weighted to correct for potential survey bias due to stratified sampling. The data obtained from the completed questionnaires were analysed using Statistical Package for Social Studies software (SPSS version 24; IBM Corporation, Armonk, NY, USA). Summary statistics including cross tabulations were obtained, where appropriate chi-squared tests were performed to assess the significant relationships between demographic variables (years since graduation, gender, and practice location) and other questions of interests, as appropriate. The level of significance was set at *p* < 0.05 (method described in detail by Lee et al., [14]).

## 3. Results

A total of 188 usable and completed questionnaires were returned, representing a response rate of 53.6%. Demographic details of the respondents were reported in the first paper of the present series [14].

### 3.1. Restorative Materials

**Figure 1:**
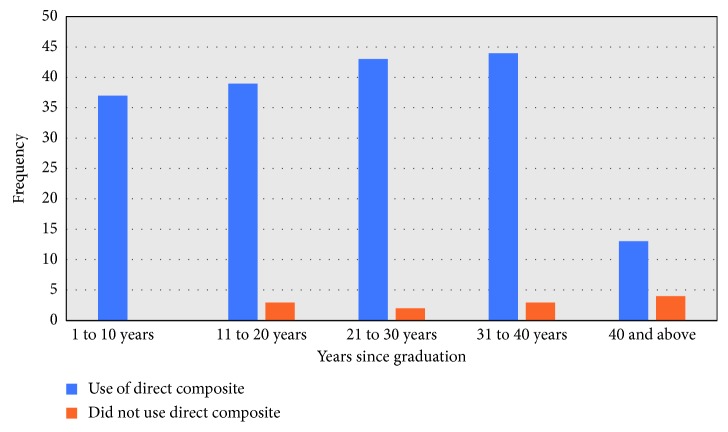
Association between years since graduation and the use of direct resin composite.

Direct resin composite was the preferred material to restore occlusoproximal class II cavities in premolars (*n*=175; 93.7%) and permanent molars (*n*=159; 85.2%). Only a small proportion of dentists reported that they use amalgam for class II cavities in premolar (*n*=19; 10%) and permanent molar (*n*=46; 23.8%) teeth. The use of resin composite for direct restorations was common among dentists who graduated less than ten years ago, compared to those who graduated 31+ years ago. This result was statistically significant in the chi-square analysis (*X* ^2^ = 10.918; *p* < 0.05) (Figure 1).

The majority of the dentists were influenced by many factors as to which material to use in the restoration of occlusoproximal cavities (Table 1). The main factors were aesthetics (*n*=115; 60.1%), followed by material durability (*n*=114; 60.2), patient preference (*n*=104; 55.3%), minimal intervention dentistry concerns (*n*=104; 54.5%), and potential for occlusal loading (*n*=72; 37.6%).

### 3.2. Bleaching and Soft Tissue Aesthetics

Eighty-six percent (*n*=164) of the respondents indicated that they provided home-based external bleaching to their patients, and 43.3% (*n*=80) of dentists provided practice-based bleaching. The common side effects experienced by patients for both types of bleaching procedures are presented in Table 2.

More than half of the surveyed dentists (*n*=103; 54%) suggested that facial soft tissue aesthetics should be included in the practice of dentistry. Among them, 28.2% indicated that all facial soft tissue aesthetics should be included within the practice scope of dentistry. However, 21.8% of the respondents thought it should be restricted to circumoral areas.

### 3.3. Endodontics

The obturation techniques used by the dentists surveyed in this study are summarised in Table 3.

**Figure 2:**
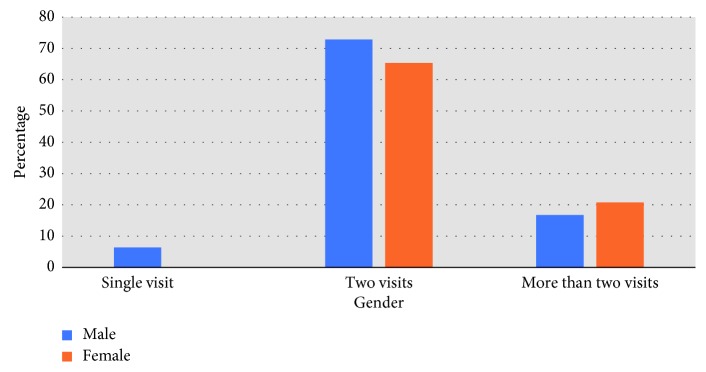
Association between gender and visits taken to complete root canal treatment.

The majority of dentists used a combination of manual and rotary instruments for cleaning and shaping root canals (*n*=67; 125%). Sixty percent of the dentists (*n*=113) reported taking two visits to complete root canal treatments for vital anterior, vital posterior (*n*=101; 53.8%), nonvital anterior (*n*=132; 70.3%), and nonvital posterior (*n*=85; 45.6%) teeth. Compared to male dentists, female dentists reported taking more than two visits to complete root canal treatments for nonvital anterior teeth, and this result was statistically significant (*X* ^2^ = 11.774; *p* < 0.05) (Figure 2).

Forty-two percent of the dentists indicated that they take more than three visits to finish a nonvital posterior root canal filling. A small number of dentists (14%) reported that they do not carry out endodontics in molar teeth due to it being a difficult procedure.

The majority of the dentists in this study (*n*=144; 76.7%) did not request cone beam computed tomography (CBCT) as a tool to aid in their endodontic diagnosis. However, a small proportion of the respondents reported that they sometimes request CBCT imaging (*n*=35; 18.2%). The main reason for CBCT requests was diagnosis of root resorption (*n*=11; 5.4%), calcified canals (*n*=18; 9.1%), and pain diagnosis (*n*=17; 8.2%).

The majority of respondents infrequently referred patients to a specialist for complex endodontic treatment (*n*=125; 67%), whilst 30.2% (*n*=57) referred patients for complex endodontics on a frequent basis.

### 3.4. Rubber Dam

Twelve percent of respondents (*n*=24) indicated that they do not use rubber dam. The majority of the dentists used the rubber dam for more than one treatment procedure. Eighty-three percent (*n*=155) used rubber dam for endodontic treatment, 24.3% (*n*=47) use it in the provision of operative dentistry, 22.5% (*n*=40) used it for practice-based bleaching, and 9.9% (*n*=18) used rubber dam for the application of fissure sealants (Table 4).

### 3.5. Paediatric Dentistry

About 49% (*n*=94) of the respondents reported that they have never used preformed metal crowns (PMCs) in the restoration of deciduous molars, with a further 18.7% (*n*=34) of dentists using it occasionally. The preferred material of choice for the restoration of class II primary molars was resin-modified glass ionomer (*n*=57; 34%), with glass ionomer cement and resin composite being used by 31.4% (*n*=57) and 16.1% (*n*=31) of the respondents, respectively. Amalgam was used by only 13% (*n*=24) of the respondents. Resin composite (*n*=99; 51.5%) was the preferred material for hypoplastic defects in first permanent molar teeth, followed by glass ionomer cement (*n*=45; 24.1%) and resin-modified glass ionomer cement (*n*=42; 21.6%) (Table 5).

The majority of dentists (*n*=135; 71.2%) indicated that they were aware of the Hall technique for the treatment of caries in deciduous teeth. There was a statistically significant association between time since graduation and awareness of the Hall technique (*X* ^2^ = 12.7723; *p* < 0.05). The majority of dentists who were aware of the technique were recent graduates (*n*=35; 95%), compared to dentists who graduated 40 or more years ago (*n*=11; 66.7%). However, only a small number of dentists (18.4%; *n*=36) reported that they have employed the Hall technique, and they were all from practices in urban centres.

## 4. Discussion

This study investigated aspects of direct restorative materials, endodontics, bleaching, and paediatric dentistry in general dental practice in New Zealand. The findings of this study provided valuable insight into aspects of the everyday dental practice currently undertaken in the country. The response rate obtained (53.6%) is considered adequate for limiting nonresponse bias for questionnaire-based studies, and the findings of this study could be compared to a similar study previously conducted in the UK [16, 17].

Over the last ten years, there has been a change in attitude towards the use of resin composite materials for the restoration of posterior teeth [18]. In this study, direct resin composite was the material of choice for restoring class II occlusoproximal cavities in premolar and permanent molar teeth. Comparatively, in a survey conducted to investigate the use of direct restorative materials in Australia, the use of resin composite materials was reported to have increased in 74% of respondents' practices, while 59% of respondents reported a decrease in use of amalgam over the previous five years [19]. A similar trend was observed in the UK where 90% of the respondents used amalgam for the restoration of occlusal-proximal cavities. However, this figure fell to 75% in 2008 and reduced again to 55% in 2015 [17, 20, 21]. This reinforces the belief that there has been a substantial shift in favour of composite materials, certainly within New Zealand, Australia, and UK due to its advantages over amalgam. These advantages are aesthetic, improved handling properties, and preservation of tooth tissue during the restorative procedure when compared to the placement of amalgam restorations [22, 23]. Resin composite was preferred among younger dentists (graduated <10 years) in comparison with older dentists (graduated 31+ years) for class II cavities. A similar trend was seen in Australia where a greater percentage of dentists who graduated within five years thought that composite use had increased [19]. This might reflect the fact that dental schools in New Zealand and Australia are providing up-to-date teaching with regard to modern materials and techniques that are better suited to meeting the patient needs. In addition, recent graduates tend to provide minimally invasive procedures with composites and other tooth-coloured restorative systems over traditional restorative materials, to promote and facilitate high-quality dental treatment [23].

Of the respondents, aesthetics (61%) and durability (61%) were the main factors influencing the decision as to which material to use for restoring occlusoproximal cavities. This is in agreement with an Australian study, where the majority of the respondents indicated that aesthetics demands (99%) and patients' wishes (96%) were the main influencing factors in the choice of materials [19]. This supports the view that in recent times, there has been an increasing demand for aesthetically appealing and minimally invasive restorations [24, 25].

Aesthetics is of great importance to many patients. In comparison with other restorative modalities, bleaching or tooth whitening is currently the least expensive and effective treatment for discoloured teeth [26]. The present study showed that 86% of the respondents provided home-based bleaching to their patients, while 43.3% provided in-office vital bleaching. These findings are in agreement with a similar study previously conducted in the UK in 2008, where home-based bleaching and in-office bleaching were provided by 90% and 28% of the dentists, respectively [17]. Home vital bleaching is common mainly because of its low cost and less tooth sensitivity compared to in-office vital bleaching [27]. There is an enormous public demand for improved aesthetics, and this has made tooth whitening a popular and often requested dental procedure despite the continuing regulatory uncertainties [28]. Patients' desires to have their teeth whitened have grown in recent years, and numerous studies suggest that tooth colour is a significant factor in the attractiveness of a smile [29, 30].

The majority of the dentists reported tooth sensitivity as the most common side effect for both types of bleaching. Several studies have reported that tooth sensitivity occurs due to the freely diffusible nature of the materials used (carbamide peroxide and hydrogen peroxide) [28, 29, 31]. The by-products of these materials can pass through the dentinal tubules and reach the pulp, causing reversible pulpitis, which results in tooth sensitivity [26, 32]. In contrast, only a small proportion of dentists in New Zealand reported soft tissue inflammation as a side effect of bleaching. Soft tissue inflammation occurs as a result of improperly fitted trays, improper or excess application of the gel, and the use of gel longer than prescribed [31]. However, no perceived systemic or long-term effects were reported by the dentists who responded to this survey.

Given the fact that an increasing number of patients are requesting aesthetic dental procedures, it does not come as a surprise that the majority of the respondents thought that facial soft tissue aesthetics should be considered a part of the scope of dentistry. Presently, *Botulinum* toxin facial injectable therapies (Botox) and dermal filler therapy are used for soft tissue augmentation to correct facial defects such as wrinkles, thin lips, and asymmetrical facial appearance [6]. However, these procedures require delivering profound anaesthesia [33]. Therefore, in order to develop clinical and aesthetic skills, it is important that dentists receive high-quality evidence-based training in the necessary techniques. Future studies should investigate whether dentists in New Zealand are performing facial aesthetic treatments to their patients and if they have accessed the recommended training to do so.

Only a few of the surveyed dentists in New Zealand (12.1%) opted for not using rubber dam. This was very similar to the UK where 13% did not use rubber dam at all [17]. The majority of the dentists in New Zealand used rubber dam mainly for endodontic treatments. A study conducted by Koshy and Chandler showed that 57% of dentists in New Zealand used rubber dam routinely in endodontic procedures [34]. This is because the use of rubber dam promotes control of cross-infection, protection, and improvement of treatment efficiency during root canal therapy [35]. However, the reported use of rubber dam for operative dentistry and bleaching was much lower (Table 4). A previous survey which investigated the use of rubber dam during operative dentistry in the United States suggested that rubber dam use is associated with dentists and patient preferences and also with restoration-level characteristics [36].

Cold lateral condensation (CLC) was the preferred obturation technique for root canal therapy in this study, which is similar to the findings previously reported in the UK [16]. Cold lateral condensation is the most widely taught and practiced obturation technique for root canal therapy because of its controlled placement of gutta-percha (GP) in the root canal and low cost of treatment [37]. Few dentists indicated that they preferred warm vertical condensation. Although this technique can be more effective in filling lateral canals compared to CLC, it requires investment in additional equipment to down-pack and backfill the canals. This might have contributed towards the limited use of this technique among dentists in New Zealand. In contrast to a previous study where 32% of the dentists used reamers for root canal shaping, rather than files or rotary instruments [16], the majority of dentists in New Zealand used a combination of both manual and rotary instrumentation for cleaning and shaping root canals.

A further aspect of the findings in respect to root canal therapy was the fact that the majority of the dentists took two or more appointments to complete root canal treatment (RCT) for vital and nonvital anterior and posterior teeth. Even though recent evidence suggests that a single visit RCT is equally successful as multiple visit RCTs [38], the majority of the respondents took more than one appointment for root canal treatment for both anterior and posterior teeth. This could possibly be explained because the surveyed dentists were general dentists who may require more time to complete the chemomechanical preparation than the trained endodontists, or the respondents would not be familiar with the evidence to support single visit treatments.

In fields of dentistry where 3D imaging is necessary, cone beam computed tomography (CBCT) is considered by some as the gold standard of imaging [39]. With respect to endodontics, CBCT provides accurate anatomical 3D images of the teeth and surrounding dentoalveolar structures, which cannot be provided by intraoral and panoramic imaging [39, 40]. The findings of this study showed that 76.7% of the dentists did not routinely request a CBCT as a tool in their endodontic diagnosis. A few dentists indicated that they sometimes use CBCT to diagnose endodontic problems such as root resorption and calcified canals. However, several studies have shown that the use of CBCT in endodontics should be limited to the assessment and treatment of complex conditions such as the identification of root canal system anomalies and determination of root curvature, assessment of traumatic injury, and assessment of vertical root fractures [40–42]. Cost and difficulties in access or a reluctance to treat complex cases could be some of the reasons why most dentists do not routinely request CBCT for endodontic diagnosis.

Forty-nine percent of the respondents indicated that they never used performed metal crowns (PMCs) in the restoration of deciduous carious teeth, which is similar to the UK study, where 56% of the respondents did not use PMCs [17]. This might be because the majority of the children in New Zealand receive free publicly funded dental care provided by dental therapists in community oral health clinics [43]. Tooth-coloured materials were the most popular choices for the restoration of the primary dentition in Australia, and the use of amalgam and stainless steel crowns was comparatively much lower in Australia. The most commonly used material for the restoration of occlusoproximal cavities in deciduous molars was resin-modified glass ionomer cements (RMGIC) whereas glass ionomer cements (55%) predominated in the UK while 32% of the respondents indicated that they used resin-modified glass ionomer cements [17]. Dental amalgam was selected by only 13% of the respondents, which was a similar finding to the UK study. In contrast, the majority of members of the Australasian Academy of Paediatric Dentistry reported using glass ionomer cement for restorations of Classes I and II in primary molars and citing the main reasons for choosing GIC over amalgam were aesthetics and fluoride release [44].

The Hall technique is a quick and noninvasive treatment for the management of carious primary molar teeth where decayed tissue is sealed under preformed metal crowns (PMCs) without local anaesthesia, drilling, or any carious tissue removal [18]. With respect to the findings of this survey, 71.2% of the dentists were aware of the Hall technique and the majority of them were recent graduates. This does not come as a surprise since the Hall technique was introduced only recently to clinical dentistry [18]. Interestingly, only a small proportion of NZ dentists reported that they used the Hall technique, despite its advantages over conventional restorative techniques [18].

## 5. Conclusions

The findings of this study highlighted that dentistry is an ever-changing profession with the introduction of refined and enhanced materials. It can be concluded from the data that general dentists in New Zealand and Australia practice similarly. Studies of this type provide a valuable insight into the practicing arrangements of dentists in New Zealand and give the opportunity of further investigating the challenges and emerging trends in general dental practice.

## Figures and Tables

**Table 1:** Factors influencing the decision on what material to use in the restoration of class II cavities.

Factor	Frequency (*n*)	Weighted percent
Aesthetics	115	60.1
Material durability	114	60
Patience preference	104	55.3
Minimal intervention dentistry concerns	104	54.5
Potential occlusal loading	72	37.6
Patient concern about mercury toxicity	56	29.7
Subsequent opportunity to refurnish and repair the restoration	23	11.8
Environmental concerns	15	18.2

**Table 2:** Main side effects experienced during bleaching.

Side effects	Home-based bleaching		Practice-based bleaching	
	Frequency (*n*)	Weighted percent	Frequency	Weighted percent
Soft tissue inflammation	34	18.2	24	13.3
Tooth sensitivity	140	74.4	64	34.9
Systemic effects	24	11.7	—	—

**Table 3:** Obturation techniques used by New Zealand dentists.

Technique	Actual frequency (*n*)	Weighted percent
Cold lateral condensation of GP	108	55.8
Warm lateral condensation of GP	29	16.0
Thermafil	21	11.7
Do not perform endodontics	10	5.8
Both cold and warm lateral condensation	10	5.0
Vertical condensation	5	2.9
Endoreze cement and GP	2	1.1
Single cone	3	1.7

**Table 4:** Rubber dam use in different dental procedures.

Procedure	Frequency (*n*)	Weighted percent
Endodontics	155	83.2
Operative dentistry	47	24.3
Practice-based bleaching	40	22.5
Fissure sealing	18	9.9

**Table 5:** Materials used for the restoration of hypoplastic defects in first permanent molar teeth.

Material	Frequency (*n*)	Weighted percent
Resin composite	99	51.5
Glass ionomer cement	45	24.1
Resin-modified glass ionomer	42	21.6
Stainless steel	11	5.9
Amalgam	10	5.3
Compomer	7	3.5

## Data Availability

The frequency and percentage data used to support the findings of this study are included within the article.

## Supplementary Materials

Supplementary Materials A detailed description of the methods is attached. Refer Section 2 in the manuscript.

## References

[1] Forrest J. L. (2009). Introduction to the basics of evidence-based dentistry: concepts and skills. *Journal of Evidence Based Dental Practice*.

[2] Gillette J. (2008). Evidence-based dentistry for everyday practice. *Journal of Evidence Based Dental Practice*.

[3] Gillette J. (2009). Striving for excellence with evidence-based dentistry. *Journal of Evidence Based Dental Practice*.

[4] CG S. (1987). Modern dentistry and the esthetically aware patient. *Journal of the American Dental Association*.

[5] Pine C. M., Pitts N. B., Steele J. G., Nunn J. N., Treasure E. (2001). Adult dental health survey: dental restorations in adults in the UK in 1998 and implications for the future. *British Dental Journal*.

[6] Dastoor S. F., Misch C. E., Wang H.-L. (2007). Dermal fillers for facial soft tissue augmentation. *Journal of Oral Implantology*.

[7] Hargreaves K. M., Berman L. H. (2015). *Cohen’s Pathways of the Pulp*.

[8] Torabinejad M., Fouad A., Walton R. E. (2014). *Endodontics-E-Book: Principles and Practice*.

[9] Nair P. N. R. (2006). On the causes of persistent apical periodontitis: a review. *International Endodontic Journal*.

[10] Kim S., Kratchman S. (2006). Modern endodontic surgery concepts and practice: a review. *Journal of Endodontics*.

[11] Glickman G. N., Koch K. A. (2000). 21st-century endodontics. *Journal of the American Dental Association*.

[12] Carrotte P. (2004). Endodontics: part 1 the modern concept of root canal treatment. *British Dental Journal*.

[13] Berg J. H. (1998). The continuum of restorative materials in pediatric dentistry-a review for the clinician. *Pediatric Dentistry*.

[14] Lee R., Ratnayake J., Veerasamy A., Loch C., Cathro P., Brunton P. A. (2018). Demographics, practicing arrangements and standards: survey among New Zealand dentists. *International Journal of Dentistry*.

[15] Brunton P. A., Ratnayake J., Loch C., Veerasamy A., Cathro P., Lee R. (2019). Indirect restorations and fixed prosthodontics: materials and techniques used by general dentists of New Zealand. *International Journal of Dentistry*.

[16] Brunton P. A., Burke F. J. T., Sharif M. O. (2012). Contemporary dental practice in the UK in 2008: aspects of direct restorations, endodontics and bleaching. *British Dental Journal*.

[17] Trevor Burke NHFW F.J., Brunton P. A. (2018). Siobhan Creanor dental practice in the UK in 2015/2016: part 4: changes since 2002?. *British Dental Journal*.

[18] Rosenblatt A. (2008). The hall technique is an effective treatment option for carious primary molar teeth. *Evidence-Based Dentistry*.

[19] Burke F. J., McHugh S., Randall R. C., Meyers I. A., Pitt J., Hall A. C. (2004). Direct restorative materials use in Australia in 2002. *Australian Dental Journal*.

[20] Brunton P. A., Sharif M. O., Creanor S., Burke F. J. T., Wilson N. H. F. (2012). Contemporary dental practice in the UK in 2008: indirect restorations and fixed prosthodontics. *British Dental Journal*.

[21] Brunton P. A., Christensen G. J., Cheung S. W., Burke F. J. T., Wilson N. H. F. (2005). Contemporary dental practice in the UK: indirect restorations and fixed prosthodontics. *British Dental Journal*.

[22] Brunthaler A., König F., Lucas T., Sperr W., Schedle A. (2003). Longevity of direct resin composite restorations in posterior teeth: a review. *Clinical Oral Investigations*.

[23] Lynch C. D., McConnell R. J., Wilson N. H. F. (2006). Challenges to teaching posterior composites in the United Kingdom and Ireland. *British Dental Journal*.

[24] Millar B. J. (2014). *Principles and Practice of Esthetic Dentistry-E-Book: Essentials of Esthetic Dentistry*.

[25] Tin-Oo M. M., Saddki N., Hassan N. (2011). Factors influencing patient satisfaction with dental appearance and treatments they desire to improve aesthetics. *BMC Oral Health*.

[26] Kihn P. W. (2007). Vital tooth whitening. *Dental Clinics of North America*.

[27] Carey C. M. (2014). Tooth whitening: what we now know. *Journal of Evidence Based Dental Practice*.

[28] Tredwin C. J., Naik S., Lewis N. J., Scully C. (2006). Hydrogen peroxide tooth-whitening (bleaching) products: review of adverse effects and safety issues. *British Dental Journal*.

[29] Dunn W. J., Murchison D. F., Broome J. C. (1996). Esthetics: patients’ perceptions of dental attractiveness. *Journal of Prosthodontics*.

[30] Grosofsky A., Adkins S., Bastholm R. (2003). Tooth color: effects on judgments of attractiveness and age. *Perceptual and Motor Skills*.

[31] Haywood V. B., Caughman W. F., Frazier K. B., Myers M. L. (2001). Tray delivery of potassium nitrate-fluoride to reduce bleaching sensitivity. *Quintessence International*.

[32] Goldberg M., Grootveld M., Lynch E. (2010). Undesirable and adverse effects of tooth-whitening products: a review. *Clinical Oral Investigations*.

[33] Cheng C. M. (2007). Cosmetic use of botulinum toxin type A in the elderly. *Clinical Interventions in Aging*.

[34] Koshy S., Chandler N. P. (2002). Use of rubber dam and its association with other endodontic procedures. *New Zealand Dental Journal*.

[35] Ahmad I. A. (2009). Rubber dam usage for endodontic treatment: a review. *International Endodontic Journal*.

[36] Gilbert G. H. L. M., Pihlstrom D. J., Amundson C. W., Gordan V. V., DPBRN Collaborative Group (2010). Rubber dam use during routine operative dentistry procedures: findings from the dental PBRN. *Operative Dentistry*.

[37] Peng L., Ye L., Tan H., Zhou X. (2007). Outcome of root canal obturation by warm gutta-percha versus cold lateral condensation: a meta-analysis. *Journal of Endodontics*.

[38] Sathorn C., Parashos P., Messer H. H. (2005). Effectiveness of single- versus multiple-visit endodontic treatment of teeth with apical periodontitis: a systematic review and meta-analysis. *International Endodontic Journal*.

[39] Scarfe W. C., Levin M. D., Gane D., Farman A. G. (2009). Use of cone beam computed tomography in endodontics. *International Journal of Dentistry*.

[40] Farman A. G. (2006). Image guidance: the present future of dental care. *Practical Procedures and Aesthetic Dentistry*.

[41] Bhuva B., Barnes J. J., Patel S. (2011). The use of limited cone beam computed tomography in the diagnosis and management of a case of perforating internal root resorption. *International Endodontic Journal*.

[42] Bernardes R. A., de Moraes I. G., Húngaro Duarte M. A., Azevedo B. C., de Azevedo J. R., Bramante C. M. (2009). Use of cone-beam volumetric tomography in the diagnosis of root fractures. *Oral Surgery, Oral Medicine, Oral Pathology, Oral Radiology, and Endodontology*.

[43] Boyd D. H., Page L. F., Thomson W. M. (2017). The Hall Technique and conventional restorative treatment in New Zealand children’s primary oral health care—clinical outcomes at two years. *International Journal of Paediatric Dentistry*.

[44] Tran L., Messer L. B. (2008). Clinicians choices of restorative materials for children. *Australian Dental Journal*.

