# Supplementary material for: Direct Restorations, Endodontics, and Bleaching: Materials and Techniques Used by General Dentists of New Zealand
Source: Int J Dent. 2019 Mar 7;2019:6327171. doi: 10.1155/2019/6327171 (PMC6431498; doi:10.1155/2019/6327171)
Supplement: Supplementary Materials — A detailed description of the methods is attached. Refer Section 2 in the manuscript. [file 6327171.f1.docx]

**Method**

Ethical approval was obtained from the University of Otago Human Ethics committee (approval number D16/098). A cross-sectional survey was conducted among general dentists practicing in New Zealand who were holding a current annual practicing certificate (APC) from the Dental Council of New Zealand in 2016. From 2131 dentists registered, contact details (email addresses, postal addresses, telephone and fax numbers) were available for only 1579 of them. From those, a subsample of 351 general dentists was selected at random from the register. Sampling was done proportionally to the number of registered dentists in each of the 14 NZ regions. A covering letter, paper copy of the questionnaire, consent form, envelope to return the completed survey form and $5 coffee voucher were distributed in 2016 to the selected participants. The Questionnaire used in this study was previously validated in a similar UK based study (4, 7). The current questionnaire was modified to suit New Zealand dental practitioners and was piloted, prior to distribution amongst 10 New Zealand dental practitioners. The questionnaire comprised of 19 sections and 125 questions (the questionnaire is available on request from the corresponding author). Questions were based on those used in an annual survey of dentists in the USA by the Clinical Research Associates and also in a similar UK-based study (7). It covered a variety of topics, including General practising information; Preventive dentistry; Restorative/Operative dentistry; Paediatric dentistry. Exclusion criteria for this study included general dentists working in universities and government departments; retired from clinical practice and general dentists who did not hold a current practising certificate.

An e-mail reminder was sent four weeks after the questionnaire was sent to all the non-respondents. Data was analysed using Statistical Package for Social Studies software (SPSS version 24; IBM Corporation, Armonk, NY, USA). Summary statistics (mean and standard deviations) were presented as appropriate for each question. Cross tabulations and chi-squared tests were used to assess the statistical association between a number of demographic variables and other questions of interest. The level of significance was set at p < 0.05.
